# A Comparison of Prognostic Factors in a Large Cohort of In-Hospital and Out-of-Hospital Cardiac Arrest Patients

**DOI:** 10.3390/life14030403

**Published:** 2024-03-18

**Authors:** Rossana Soloperto, Federica Magni, Anita Farinella, Elisa Gouvea Bogossian, Lorenzo Peluso, Nicola De Luca, Fabio Silvio Taccone, Filippo Annoni

**Affiliations:** 1 Department of Intensive Care, Brussels University Hospital (HUB), Free University of Brussels (ULB), 1070 Brussels, Belgium; rossana.soloperto@gmail.com (R.S.); federica.magni24@gmail.com (F.M.); anita.farinella92@gmail.com (A.F.); elisagobog@gmail.com (E.G.B.); lorenzopeluso80@gmail.com (L.P.); filippo.annoni@erasme.ulb.ac.be (F.A.); 2 Department of Interdisciplinary Medicine, Intensive Care Unit Section, “Aldo Moro” University, 70124 Bari, Italy; 3 Emergency Department, ASST Sette Laghi, Ospedale di Circolo e Fondazione Macchi, 21100 Varese, Italy; 4 Department of Anesthesia and Intensive Care, Istituto Mediterraneo per I Trapianti e Terapie ad alta Specializzazione (IRCCS-ISMETT), 90127 Palermo, Italy; 5 Hypertension Research Center, Department of Advanced Biomedical Science, “Federico II” University of Naples, 80131 Naples, Italy; nideluca@unina.it; 6 Experimental Laboratory of Intensive Care, Free University of Brussels (ULB), 1070 Brussels, Belgium

**Keywords:** heart arrest, prognosis, predictors, neurological outcome

## Abstract

We investigated independent factors predicting neurological outcome and death, comparing in-hospital (IHCA) and out-of-hospital cardiac arrest (OHCA) patients. The study was conducted in the mixed 34-bed Intensive Care Department at the Hôpital Universitaire de Bruxelles (HUB), Belgium. All adult consecutive cardiac arrest (CA) survivors were included between 2004 and 2022. For all patients, demographic data, medical comorbidities, CA baseline characteristics, treatments received during Intensive Care Unit stay, in-hospital major complications, and neurological outcome at three months after CA, using the Cerebral Performance Category (CPC) scale, were collected. In the multivariable analysis, in the IHCA group (*n* = 540), time to return of spontaneous circulation (ROSC), older age, unwitnessed CA, higher lactate on admission, asystole as initial rhythm, a non-cardiac cause of CA, the occurrence of shock, the occurrence of acute kidney injury (AKI), and the presence of previous neurological disease and of liver cirrhosis were independent predictors of an unfavorable neurological outcome. Among patients with OHCA (*n* = 567), time to ROSC, older age, higher lactate level on admission, unwitnessed CA, asystole or pulseless electrical activity (PEA) as initial rhythm, the occurrence of shock, a non-cardiac cause of CA, and a previous neurological disease were independent predictors of an unfavorable neurological outcome. To conclude, in our large cohort of mixed IHCA and OHCA patients, we observed numerous factors independently associated with a poor neurological outcome, with minimal differences between the two groups, reflecting the greater vulnerability of hospitalized patients.

## 1. Introduction

Sudden cardiac arrest (CA) ranks as the third leading cause of death in Europe, with significant variations in incidence and outcomes between and within countries [1,2]. Out-of-hospital cardiac arrest (OHCA) occurs at a rate of 67 to 170 per 100,000 people annually, while in-hospital cardiac arrest (IHCA) ranges from 1.5 to 2.8 per 1000 hospital admissions. Despite advances in pre-hospital and in-hospital management, survival rates remain poor, with only 11.7% of patients surviving to discharge [2]. Moreover, only a minority of patients experience full neurologic recovery, with many survivors presenting persistent long-term physical and cognitive disabilities [3,4].

Prognostication of CA thereby remains crucial in this setting, to allow prompt identification of patients with poor neurological outcomes. However, while a multimodal neuro-prognostication strategy is recommended [2], there is no single predictive tool that is sufficiently accurate alone and the availability of such tools (i.e., electrophysiological tests, brain imaging, automated pupillometry, serum biomarkers) is extremely variable among centers. Several factors can affect survival after CA; specifically, in OHCA patients, gender, the cause of arrest, the initial arrest rhythm, comorbidities, event location, and socioeconomic state might all have a role as baselines characteristics in determining patients’ outcomes [5,6], along with post-resuscitation care with the control of oxygenation [7] and early ventilator settings [8]. In IHCA patients, age, gender, comorbidities, and the underlying disease are significantly associated with a poor outcome [9,10,11]. Moreover, in both cohorts, as reported in a recent meta-analysis by Sanfilippo et al., target temperature management (TTM) at 32–34 °C was associated with higher survival when compared to “uncontrolled” normothermia (RR: 1.31 (95% CI 1.07, 1.59), *p* = 0.008) [12].

Among the limitations of the current multimodal neuro-prognostication approach, it should be considered that one-third of patients admitted to the Intensive Care Unit (ICU) following OHCA and up to two-thirds of IHCA patients die due to non-neurological injury [13,14]. Additionally, predicting patient outcomes requires a minimum of 72 h of observation, while intensive and aggressive therapies might not be effective in those patients with severe reperfusion injury and pre-existing frailty.

As such, research focusing on immediately available factors that predict mortality would inform about the severity and degree of risk of admitted patients. However, although several studies have assessed such issues in OHCA or IHCA populations [15,16,17,18] there is scarce literature [19] on the comparisons of predictive factors in cohorts combining IHCA and OHCA patients from the same institutions who would receive a similar therapeutic and prognostic approach, reducing the effects of heterogeneity in practices on measured outcomes.

The aim of this study was therefore to assess and compare the predictive factors of a poor neurological outcome between IHCA and OHCA patients.

## 2. Materials and Methods

### 2.1. Study Population

This study was conducted in the 34-bed medical–surgical Intensive Care Unit at Hôpital Universitaire de Bruxelles (HUB), in Brussels, Belgium. All consecutive patients aged over 18 years admitted after IHCA or OHCA of non-traumatic cause who achieved return of spontaneous circulation (ROSC) following cardiopulmonary resuscitation (CPR) were retrospectively included in an institutional database. We analyzed data from all patients admitted from January 2004 to December 2021. Patients with missing data on neurological outcome were excluded.

### 2.2. Data Collection

Post-resuscitation care adhered to an institutional protocol in accordance with current guidelines at that time and evidence from previous studies [20,21]. We collected demographic data for all patients, including age, sex at birth, and weight, as well as primary medical comorbidities (i.e., arterial hypertension, chronic heart failure, COPD/asthma, coronary artery disease, diabetes, chronic kidney disease, liver cirrhosis, HIV, and previous neurological disability). CA baseline characteristics were recorded: witness of CA, bystander CPR, epinephrine dose administered during CPR, time to ROSC, location (in-hospital or out-of-hospital CA), cause of CA (cardiac origin for acute coronary syndrome and/or cardiac arrhythmia vs. non-cardiac causes, such as respiratory, neurological, or miscellaneous causes), first recorded heart rhythm (shockable or non-shockable), and initial lactate level on admission.

Treatments received during Intensive Care Unit stay were also recorded and included use of extracorporeal cardiopulmonary resuscitation (ECPR) or veno-arterial extracorporeal membrane oxygenation (VA-ECMO), application of intra-aortic balloon pump counterpulsation (IABP), target temperature management, continuous renal replacement therapy (CRRT), and administration of steroids. Additionally, complications during the hospital stay, such as acute kidney injury, defined according to KDIGO criteria [22], the occurrence of shock from any cause and any hemorrhagic events were also retrieved from the institutional database.

### 2.3. Study Outcomes

The primary objective of the study was to compare the predictive variables of unfavorable neurological outcome (UO) between IHCA and OHCA patients at three months. Secondary outcomes included the difference in predictive variables for mortality at 90 days between IHCA and OHCA patients and the assessment of independent predictors of a UO in the overall population.

Neurological outcome was assessed at three months after CA, using the Cerebral Performance Category (CPC) scale [23]. For this study, the CPC scale was dichotomized as unfavorable (UO, CPC 3–5) and favorable outcomes (FO, CPC 1–2). Data were collected through electronic reports of neurological medical examinations conducted during follow up at three months post-CA.

For patients who died in the Intensive Care Unit, the cause of death was determined and defined as related to “non-neurological causes” (i.e., when death occurred as a direct consequence of shock, including subsequent multiorgan failure) or “neurological causes” (i.e., if this led to withdrawal of life-sustaining treatment or brain death). All data were collected from the patient data management software in the Intensive Care Unit (PDMS, Picis Critical Care Manager, Picis Inc., Wakefield, MA, USA).

### 2.4. Statistical Analysis

R version 4.3.1 was used as statistical software for the analysis. Descriptive statistics were expressed as frequencies and percentages for categorical variables. The central tendency of continuous variables was characterized using means with standard deviations for normally distributed variables (as determined by the Kolmogorov–Smirnov test, with sample size *n* > 100), and using medians with interquartile ranges (IQRs) for non-normally distributed variables. To evaluate all variables predictive of a UO, separate logistic multivariate analyses were conducted for each population; a multivariate analysis was subsequently performed for the combined population, with OHCA as a covariate in the model, as well as the variable time to account for potential confounders; this approach aimed to verify whether changes in CA management over time due to guideline updates or training impacted outcomes.

For univariate correlation analyses, categorical variables were compared using the chi-square test and Fisher’s exact test when appropriate. Bonferroni’s pairwise correction for overall *p*-value was applied to all categorical variables with multiple levels. Continuous variables were compared using one-way ANOVA for normally distributed variables and the nonparametric Mann–Whitney U test for non-normally distributed variables. Logistic univariate regression assessed the association of demographic characteristics, baseline CA characteristics, and hospital stay complications, and provided pharmacological and invasive treatments with a UO. The Wald test obtained the overall *p*-value for categorical variables with more than two levels. For the logistic multivariable model analysis, factors with a *p*-value of less than 0.1 in univariate analyses were considered for inclusion. A percentage of missing variables under 10% was deemed acceptable. Age and gender variables were considered as confounders and included in the multivariable analysis.

To provide a clinically useful cut-off for all significant continuous variables, the optimal threshold for discriminating between FO and UO was determined through ROC curve analyses for IHCA, OHCA, and combined populations. Backward–forward stepwise selection was applied using the likelihood ratio test with Akaike’s information criterion (AIC) as the stopping rule. Multivariate logistic model performance was evaluated by calculating the area under the receiver operating characteristic (ROC) curve for IHCA, OHCA, and the combined population, enabling the assessment of the model’s discrimination performance. The Hosmer–Lemeshow goodness-of-fit test (using deciles of estimated probability) was used to assess model fit.

A correlation matrix based on Pearson’s chi-square coefficient for normally distributed variables and Spearman’s rank correlation coefficient for non-normally distributed variables was used to check for collinearity in independent variables before undertaking multivariate analysis. A correlation coefficient value > 0.3 was considered the cut-off for significant multicollinearity. In cases of multicollinearity, the causal variable was included in the model, or the variable that best explained the model in terms of the estimate β regression coefficient was selected when no causality could be established. Lastly, in the overall population, a Cox proportional hazard regression analysis was performed for three-month mortality outcomes. Variables with a *p*-value < 0.1 in univariate Cox regression were included in the multivariate Cox model. The final Cox regression model was based on stepwise backward selection according to AIC; the assumption of proportional risk and log-rank test were verified. The sum of all risk factors identified in the Cox regression model was plotted in a Kaplan–Meier (KM) survival curve. For the “site of arrest” variable, a KM curve was performed, adjusted for all other significant covariates at Cox regression. A value of *p* < 0.05 was retained as statistically significant.

All statistical analyses were performed using the statistical software R (R version 4.2.2). As this is an analytical retrospective study with all consecutive patients admitted for CA enrolled, sample size calculation was not performed.

## 3. Results

### 3.1. Study Population

A total of 1123 patients were admitted after CA over the study period. Of those, 16 (0.1%) patients were excluded due to missing neurological outcomes, leaving 1107 patients for the final analysis. The characteristics of the study population are shown in Table 1.

Patients with IHCA were younger, presented more comorbidities, and more frequently experienced a witnessed CA and underwent bystander CRP than OHCA patients. A non-cardiac cause of CA was more frequent and mean time to ROSC was significantly shorter in IHCA than in OHCA patients. Urgent percutaneous coronary intervention was significantly less frequent in the IHCA population compared to the OHCA population. Complications during hospital stay (such as need for CRRT, hemorrhagic events, infections, AKI, shock) were significantly more frequent in the IHCA population than in the OHCA population, while the retained cause of death was non-neurological in most IHCA patients (58%) and neurological in most OHCA patients (70.5%).

### 3.2. Primary Outcome

#### 3.2.1. Neurological Outcome in IHCA Patients

In IHCA patients, 372 (68.9%) had a UO; of those, 16 (4.3%) had a CPC of 3 or 4. IHCA patients with a UO were significantly older, less frequently had a witnessed CA, less frequently received bystander CPR, and were less likely to have an initial shockable rhythm than patients with a FO. The main differences between a UO and FO after IHCA are presented in Supplemental Tables S1 and S2.

In the multivariable logistic regression model, longer time to ROSC, older age, unwitnessed arrest, higher lactate on admission, asystole as initial rhythm, a non-cardiac cause of CA, the occurrence of shock, the occurrence of AKI, and the presence of previous neurological disease and of liver cirrhosis were independent predictors of a UO (Table 2).

**Figure 1:**
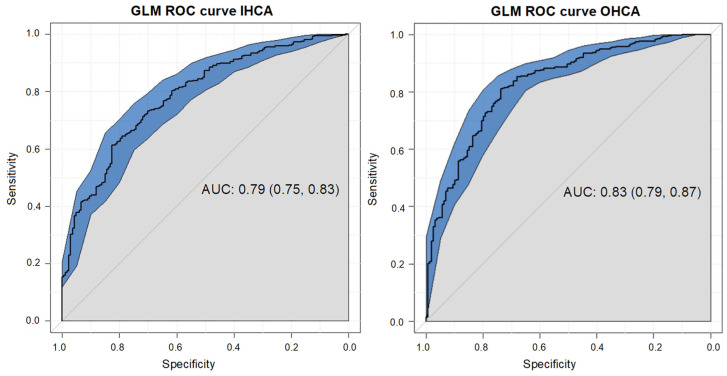
ROC curve for logistic multivariate model for IHCA and OHCA population, respectively. The ROC curve for IHCA to assess the discriminatory ability of the model was 0.79 [95% CI 0.75–0.83]. The ROC curve for OHCA to assess the discriminatory ability of the model was 0.83 [95% CI 0.79–0.87].

The ROC curve to assess the discriminatory ability of the model to predict a UO was 0.79 [95% CI 0.75–0.83], as shown in Figure 1. The best cut-offs for time to ROSC, age, and lactate on admission to predict a UO were 9.5 min, 61.5 years, and 5.95 mmol/L (Supplemental Figure S1).

#### 3.2.2. Neurological Outcome in OHCA Patients

In OHCA patients, 407 (71.8%) patients had a UO; of those, 16 had CPC of 3–4. Patients with a UO were older, less frequently had a witnessed CA and bystander CPR, were less likely to have had an initial shockable rhythm, and had a longer time to ROSC and higher lactate on admission. The main differences between a UO and FO after OHCA are presented in Supplemental Tables S3 and S4.

In the multivariable logistic regression model, longer time to ROSC, older age, higher lactate levels on admission, unwitnessed CA, asystole or pulseless electrical activity (PEA) as initial rhythm, the occurrence of shock, a non-cardiac cause of CA, and a previous neurological disease were independent predictors of a UO (Table 2).

The ROC curve to assess the discriminatory ability of the model to predict a UO was 0.83 [95% CI 0.79–0.87], as shown in Figure 1. The optimal cut-offs for time to ROSC, age, and lactate on admission to predict a UO were 19.5 min, 63.5 years, and 6.5 mmol/L (Supplemental Figure S2).

### 3.3. Secondary Outcomes

Among patients with IHCA, 356 (65.9%) died at 3 months; among these patients, 349 (98.0%) died during the hospital stay, of which, 317 (89.0%) died in the ICU. The cause of death was determined only for patients who died in the ICU (*n* = 317); 133 (43%) had a neurological and 184 (58%) had a non-neurological cause of death.

Among patients with OHCA, 391 (69.0%) patients died at 3 months; among them, 388 (99.2%) died during the hospital stay, of which, 380 (97.9%) died in the ICU. The cause of death was determined only for patients who died in the ICU (*n* = 380); 268 (62%) patients had a neurological and 112 had a non-neurological cause of death (*p* < 0.001 vs. IHCA).

**Figure 2:**
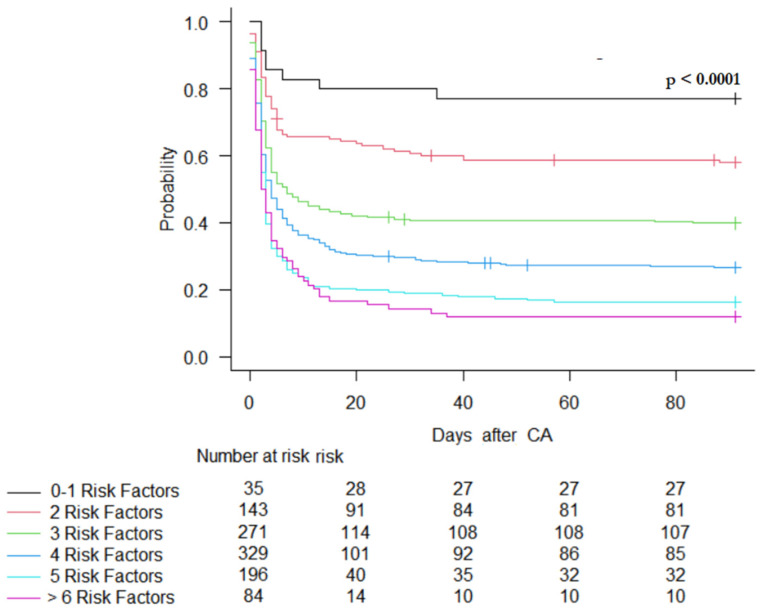
Kaplan–Meier plot of survival at 90 days, according to the number of predictors. Risk factors identified in Cox hazard regression model for overall population: lactate on admission ≥ 6.75; previous neurological disability; unwitnessed CA; occurrence of shock; time to ROSC ≥ 13.5 min; age ≥ 63.5 years, no need for urgent percutaneous coronary intervention; initial non-shockable rhythm.

In the Cox proportional hazard regression model, high lactate on admission, previous neurological disease, unwitnessed CA, the occurrence of shock, longer time to ROSC, older age, lack of urgent PCI, and an initial non-shockable rhythm were significantly associated with an increased risk of a UO, while the location of cardiac arrest was not (*p* > 0.05). The Kaplan–Meier curve showed that the time to event was shorter in patients with multiple predictors of mortality (log-rank test *p* < 0.001, Figure 2).

**Figure 3:**
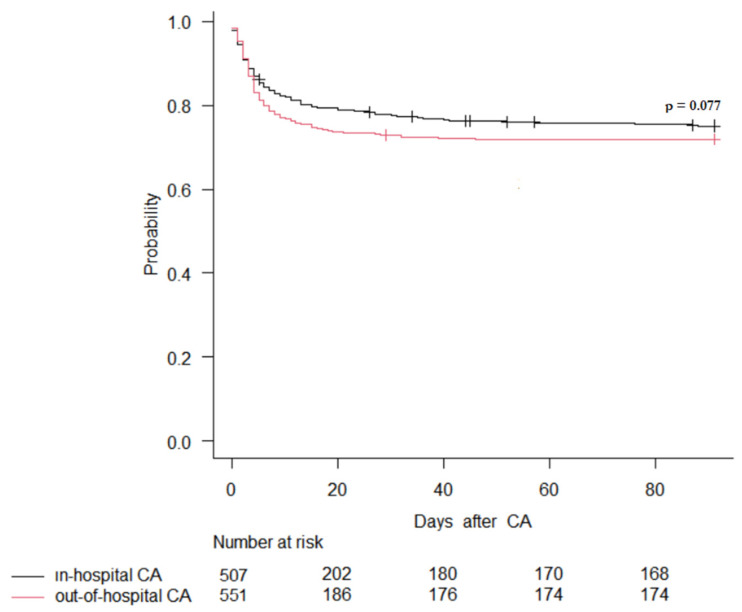
Adjusted KM Survival Curve for site of cardiac arrest. Adjusting factors: unwitnessed CA; time to ROSC; lactate on admission; age; previous neurological disability; no need for urgent percutaneous coronary intervention; initial non-shockable rhythm; occurrence of shock.

The Kaplan–Meier curve for the variable “cardiac arrest location”, adjusted for all factors found to be significant in the Cox proportional hazards analysis, showed that the cardiac arrest location had no impact on 90-day mortality (Figure 3).

Differences between patients with a UO and FO in the overall population are reported in the Supplementary Materials (Supplemental Tables S5 and S6), as are results of the multivariable analysis for predictors of UO (Supplemental Table S7) and the ROC curve assessing the discriminatory ability of the logistic model (Supplemental Figure S3).

## 4. Discussion

In this retrospective study, we observed similar predictors of a UO between IHCA and OHCA patients. Moreover, the increase in the number of predictors was associated with a shorter time to death. Earlier studies comparing IHCA and OHCA patients have varied in population size and cohort definitions, leading to differences in characteristics and outcomes. Some of these studies are older and may not reflect improvements in outcomes over the recent years [24,25]. Few studies have directly compared IHCA and OHCA cohorts admitted to ICUs [24,25,26]. Most of these studies included all patients who underwent cardiopulmonary resuscitation [24,27], with some separately analyzing patients with return of spontaneous circulation (ROSC) and those who survived until hospital discharge [24,26,28]. Only two studies have focused on patients admitted to the ICU, with one involving a selected group of patients managed with TTM [24,25].

In the current study, multivariable prediction models were developed for early prognostication in a cohort of unselected adult non-traumatic IHCA and OHCA patients admitted to the ICU after ROSC. The baseline characteristics of IHCA and OHCA populations in this study were found to be remarkably similar, supporting the notion that these populations have many commonalities. These findings align with other comparable studies conducted in the Netherlands and Sweden [19,24]. Patients admitted to the ICU after IHCA were generally older, more often female, and had more comorbidities than OHCA patients. This trend was also observed in a Danish registry study of patients with ROSC [27]. Studies involving patients managed with TTM found similar demographics between IHCA and OHCA cohorts, which could suggest selection bias or random effects due to small study sizes [25]. Higher rates of witnessed arrests and shorter delay times were consistently reported for IHCA compared to OHCA cohorts, likely reflecting in-hospital monitoring and proximity to care. In the current study, the first recorded rhythm was more often non-shockable in IHCA patients, consistent with lower rates of a cardiac cause of the arrest in this cohort. Consequently, a significantly lower percentage of IHCA patients underwent PCI compared to OHCA patients.

Previous data on first monitored rhythms have been conflicting [25,29,30], possibly due to differences in cohort definitions, critical care outreach availability, emergency medical service (EMS) systems, bystander cardiopulmonary resuscitation rates, and do-not-resuscitate (DNR) orders. Additionally, IHCA patients with shockable rhythms may have shorter delay times and not always require ICU admission, resulting in their exclusion from the study. However, in IHCA patients, more factors appear to negatively affect neurological outcomes.

In patients with IHCA, the most frequent initial rhythm observed was asystole, while the relative frequency of primary pulseless electrical activity (PEA) was higher compared to out-of-hospital cardiac arrest (OHCA). This discrepancy could be attributed to differences in etiology between the two groups. Alternatively, it is possible that PEA in OHCA patients often progresses to asystole by the time Emergency Medical Services reach the patient, whereas IHCA patients are typically discovered earlier and are thus more likely to retain PEA upon arrival of the medical team. Conversely, the trend toward more frequent ventricular fibrillation in OHCA patients may reflect a higher prevalence of coronary artery disease in this population. Treatment advantages were generally observed in IHCA patients, with higher rates of bystander cardiopulmonary resuscitation and initial care provided by healthcare professionals. However, despite better treatment of IHCA, our study indicates favorable treatment outcomes in OHCA patients compared to previous research [31,32,33], with 48.5% of OHCA patients receiving bystander CPR, a critical intervention associated with increased survival rates [34].

In OHCA patients, initial non-shockable rhythms, such as asystole, occurrence of shock after cardiac arrest, non-cardiac etiology of arrest, elevated lactate levels on admission, prolonged time to return of spontaneous circulation, and older age, were identified as independent factors associated with unfavorable outcomes. Similarly, in IHCA patients, in addition to factors observed in the OHCA population, acute kidney injury after cardiac arrest, history of neurological disease, and liver cirrhosis were independently associated with unfavorable outcomes, consistent with previous findings [35,36,37].

Furthermore, while the optimal threshold for discriminating between favorable and unfavorable outcomes regarding time to ROSC was 19.5 min for OHCA patients, it was significantly lower at 9.5 min for IHCA patients, suggesting a greater impact of no-flow or low-flow time on IHCA outcomes due to the severity in hospitalized patients.

Additionally, lactate levels on admission and age thresholds differed significantly between IHCA and OHCA patients, with higher values associated with an increased likelihood of unfavorable outcomes. In the overall population, lactate levels exceeding 6.75 mmol/L, a history of neurological disease, unwitnessed cardiac arrest, shock occurrence post-arrest, prolonged time to ROSC, older age, non-ischemic cause of arrest, and initial non-shockable rhythm emerged as significant independent predictors of 90-day mortality, consistent with the existing literature [38,39,40,41].

Furthermore, the risk of 90-day mortality increased with the number of these risk factors.

Unlike previous prognostic models necessitating information on no-flow duration [42,43], our model does not require this data, making it more universally applicable. Moreover, the location of cardiac arrest showed no significant correlation with neurological outcomes or pure survival at three months, suggesting that distinguishing between IHCA and OHCA may be less crucial now than previously assumed, given the similar outcomes at three months, as reported in a previous study [19]. Importantly, differences in short-term mortality and neurological outcomes may be influenced by hospital policies regarding do-not-resuscitate (DNR) orders and termination-of-resuscitation protocols, impacting admission to the Intensive Care Unit and patient management during ICU stays [44,45], which may vary across countries. In contrast to existing prognostic scores for cardiac arrest, such as the Cardiac Arrest Hospital Prognosis (CAHP) score, MIRACLE2, C-GRAPh, and CRASS [40,46,47,48], which are all limited to OHCA patients, our prediction model can be used for both populations. Our study offers clinicians a simple tool for early risk stratification of adverse outcomes at 3 months, instead of providing a traditional score.

Unlike models constructed from highly selected patient populations, our study included a diverse population of adult cardiac arrest survivors, regardless of arrest location, initial cardiac rhythm, or whether the patient received TTM. This broad inclusion allows our model to be applicable to most adult non-traumatic cardiac arrest patients, contrasting with previous studies that primarily focused on predictors of outcomes in either in-hospital or out-of-hospital cardiac arrest populations [3,49,50]. Although we found similar predictors of poor outcomes irrespective of arrest location, differences in underlying causes, resuscitation durations, and patient pre-existing conditions should yield significantly divergent prognoses between IHCA and OHCA. Strengths of our study include that data for both populations were collected from patients admitted to the same hospital, responsible for both pre-hospital and ICU care, ensuring similar treatment protocols despite different team compositions. Additionally, our model’s strength lies in the fact that withdrawal of life-sustaining treatment (WLST) was performed using the same prognostic algorithm, based on previous evidence [51,52] and using several prognostication modalities, in accordance with CoSTR on the prediction of poor outcome, which includes distinct recommendations [53].

Our study also has several limitations. First, it was a retrospective analysis of data from a single-center cohort. Given that patient characteristics and care processes vary across regions and countries, additional external validations should be performed to assess generalizability. Second, the relatively high OHCA survival in our region might be due to selection bias, as only patients who achieved ROSC were enrolled. However, survival rates align with previous data, suggesting the reported rate is mostly accurate. Third, for the IHCA population, arrest location and monitoring system presence were missing. Forth, information regarding CPC scores and presumed CA causes were collected from patient records, introducing the possibility of inter-observer variability. Finally, our models, based on variables available upon ICU admission, cannot be used for decision-making regarding the withdrawal of life sustaining-treatment. Current resuscitation guidelines recommend delaying prognostication until at least 72 h after ROSC to avoid decisions regarding the premature withdrawal of life-sustaining treatment [2]. However, early patient-data-driven prognosis estimates may still be useful for communication with surrogate decision-makers and guiding treatment decisions, such as offering maximal treatment and critical interventions, such as veno-arterial extracorporeal membrane oxygenation.

## 5. Conclusions

In this study, we identified comparable predictive factors of an unfavorable neurological outcomes in both in-hospital and out-of-hospital cardiac arrest patient cohorts. Nevertheless, we observed slight distinctions between the two cohorts. In particular, comorbidities and complications during ICU care seemed to exert a more pronounced influence in the IHCA population, highlighting the inherent vulnerability of hospitalized patients.

## Figures and Tables

**Table 1:** Table of characteristics of study population, according to the location of arrest (IHCA = in hospital; OHCA = out of hospital).

	Units of Measure	Variables	All (*n* = 1107)	IHCA (*n* = 540)	OHCA (*n* = 567)	*p*-Value
Demographic characteristics	Mean (SD)	Age (years)	65 (53–75)	64.51 (15.3)	61.99 (14.9)	0.006
	*n* (%)	Male sex	728 (65.8)	338 (62.6)	390 (68.8)	0.031
	Median (IQR)	Weight (Kg)	77 (66–89)	77.5 [65.0, 90.0]	77.0 [67.3, 88.0]	0.59
Comorbidities	*n* (%)	None	116 (10.5)	36 (6.7)	80 (14.1)	<0.001
	*n* (%)	Chronic heart failure	271 (24.5)	175 (32.4)	96 (16.9)	<0.001
	*n* (%)	Diabetes	266 (24.0)	161 (29.8)	105 (18.5)	<0.001
	*n* (%)	Arterial hypertension	471 (42.5)	254 (47.0)	217 (38.3)	0.004
	*n* (%)	Coronary artery disease	404 (36.5)	198 (36.7)	206 (36.3)	0.95
	*n* (%)	COPD	204 (18.4)	106 (19.6)	98 (17.3)	0.35
	*n* (%)	Chronic kidney disease	185 (16.7)	143 (26.5)	42 (7.4)	<0.001
	*n* (%)	Liver cirrhosis	53 (4.8)	36 (6.7)	17 (3.0)	0.005
	*n* (%)	HIV	6 (0.5)	4 (0.7)	2 (0.4)	0.38
	*n* (%)	Previous neurological disease	175 (15.8)	93 (17.2)	82 (14.5)	0.21
Cardiac arrest characteristics	*n* (%)	Witnessed arrest	846 (76.4)	457 (84.6)	389 (68.6)	<0.001
	*n* (%)	Bystander CRP	722 (65.2)	447 (82.8)	275 (48.5)	<0.001
	*n* (%)	Presentation rhythm				
		Shockable rhythm	438 (39.6)	183 (33.9)	255 (45.0)	<0.001
		Asystole	441 (39.8)	206 (38.1)	235 (41.4)	
		PEA	194 (17.5)	126 (23.3)	68 (12.0)	
		Unknown	34 (3.1)	25 (4.6)	9 (1.6)	
	Median (IQR)	Time to ROSC (min)	17 [10.3]	13.0 [6.0, 22.0]	20.0 [14.5, 30.0]	<0.001
	*n* (%)	Non-cardiac cause	495 (44.7)	284 (52.6)	211 (37.2)	<0.001
	Median (IQR)	Lactate on admission (mmol/L)	5.9 [3.5, 9.4]	5.60 [3.3, 9.0]	6.2 [3.8, 9.9]	0.016
		Epinephrine (mg)	3 [1–5]	3 [1–5]	3 [2–6]	<0.001
Medical diagnostics and therapeutic interventions during ICU	*n* (%)	Coronary angiography	349 (31.5)	82 (15.2)	267 (47.1)	<0.001
	*n* (%)	Corticosteroids	246 (22.2)	145 (26.9)	101 (17.8)	<0.001
	*n* (%)	CRRT	173 (15.6)	120 (22.2)	53 (9.4)	<0.001
	*n* (%)	ECMO	116 (10.5)	50 (9.3)	66 (11.6)	0.20
	*n* (%)	ECPR	88 (7.9)	42 (7.8)	46 (8.2)	0.91
	*n* (%)	Hypothermia	680 (61.4)	260 (48.1)	420 (74.1)	0.001
	*n* (%)	IABP	50 (4.5)	28 (5.2)	22 (3.9)	0.31
	*n* (%)	Dobutamine	466 (42.1)	234 (43.3)	232 (40.9)	0.43
	*n* (%)	Vasopressors	836 (75.5)	421 (78.0)	415 (73.2)	0.07
	*n* (%)	Mechanical ventilation	1107 (100)	540 (100)	567 (100.0)	NA
	*n* (%)	Steroids	44 (4.0)	34 6.3)	10 (1.8)	<0.001
	*n* (%)	Percutaneous coronary intervention	349 (31.5)	37 (6.9)	117 (20.6)	<0.001
Complications during hospital stay	*n* (%)	Hemorrhagic events	87 (7.9)	53 (9.8)	34 (6.0)	0.019
	*n* (%)	Infections	570 (51.5)	312 (57.8)	258 (45.5)	0.001
	*n* (%)	AKI	593 (53.6)	320 (59.3)	273 (48.1)	<0.001
	*n* (%)	Shock	540 (48.8)	298 (55.2)	242 (42.7)	<0.001
Outcome	*n* (%)	Death within 24 h	102 (9.2)	57 (10.6)	45 (7.9)	0.15
	*n* (%)	Death within 48 h	240 (21.7)	122 (22.6)	118 (20.8)	0.51
	*n* (%)	Death within 72 h	381 (34.4)	186 (34.4)	195 (34.4)	1.00
	*n* (%)	Death at 3 months	747 (67.5)	356 (65.9)	391 (69.0)	0.31
	*n* (%)	Death in ICU	697 (63.0)	317 (45.5)	380 (54.5)	0.005
	*n* (%)	Cause of death for patients in ICU				<0.001
		Non-neurological	296 (26.7)	184 (34.07)	112 (19.8)	
		Neurological	401 (36.2)	133 (24.63)	268 (47.3)	
		Unknown	410 (37.0)	223 (41.3)	187 (33.0)	
	*n* (%)	Hospital death	737 (66.6)	349 (64.6)	388 (68.4)	0.18
	*n* (%)	CPC at three months				0.29
		1	237 (21.4)	116 (21.5)	121 (21.3)	
		2	91 (8.2)	52 (9.6)	39 (6.9)	
		3	27 (0.5)	12 (2.2)	15 (2.6)	
		4	5 (2.4)	4 (0.7)	1 (0.2)	
		5	747 (67.5)	356 (65.9)	391 (69.0)	
	*n* (%)	Unfavorable composite outcome at 3 months	779 (70.4)	372 (68.9)	407 (71.8)	0.29

HIV: human immunodeficiency virus; CRP: cardiopulmonary resuscitation; PEA: pulseless electrical activity; ROSC: return to spontaneous circulation; CRRT: continuous renal replacement therapy; ECMO: extracorporeal membrane oxygenation; ECPR: extracorporeal cardiopulmonary resuscitation; IABP: intra-aortic balloon pump; ICU: Intensive Care Unit; AKI: acute kidney insufficiency; CPC: Cerebral Performance Category; COPD: chronic obstructive pulmonary disease.

**Table 2:** Multivariate logistic regression for IHCA and OHCA cohorts.

Factor		Final Multivariate Logistic Model Based on AIC Selection							
		IHCA Population				OHCA Population			
		OR	IC95%		*p*-Value	OR	IC95%		*p*-Value
**Witnessed CA**		0.490	0.258	0.930	0.029	0.474	0.283	0.796	0.005
**Time to ROSC (min)**		1.030	1.020	1.050	<0.001	1.030	1.010	1.050	>0.001
**Non-cardiac cause**		1.730	1.120	2.670	0.013	1.840	1.090	3.110	0.023
**Shock**		1.570	1.010	2.440	0.047	1.970	1.210	3.180	0.006
**Age (years)**		1.030	1.020	1.050	<0.0001	1.040	1.020	1.060	<0.0001
**Lactate levels (mmol/L)**		1.100	1.040	1.170	0.001	1.070	1.000	1.130	0.048
**Presentation Rhythm** **^#^**	**VF/VT**	REF				REF			
	**Asystole**	4.220	2.530	7.050	<0.0001	5.740	3.360	9.800	<0.0001
	**PEA**	1.590	0.928	2.720	0.09	3.670	1.680	8.010	0.001
**Previous neurological disease**		2.180	1.180	4.030	0.012	1.700	0.840	3.450	0.14
**Liver cirrhosis**		3.4000	1.100	10.600	0.034	-	-		-
**AKI**		1.420	0.912	2.200	0.121	-	-		-
**Null deviance**		662.01 on 531 degrees of freedom				668.22 on 559 degrees of freedom			
**Residual deviance**		534.87 on 519 degrees of freedom				496.60 on 549 degrees of freedom			
**AIC**		560.9				518.6			
**Hosmer and Lemeshow goodness of fit (GOF)**		χ^2^ = 8.6, df = 8, *p*-value = 0.4				χ^2^ = 8.4, df = 8, *p*-value = 0.4			
**AUC Model’s ROC curve**		0.787 [95% CI 0.746–0.827]				0.829 [95% CI 0.792–0.867]			

Final reduced model has been obtained after stepwise back/forward selection based on Akaike Information Criterion (AIC). ^#^ Overall *p*-value for initial rhythm: <0.0001.

## Data Availability

Data are available on request to the corresponding author and according to ethical restrictions.

## Supplementary Materials

The following supporting information can be downloaded at: https://www.mdpi.com/article/10.3390/life14030403/s1, Table S1: Univariate correlation analysis for quantitative variables in in-of-hospital cardiac arrest population; Table S2: Univariate correlation analysis for categorial variables in IHCA population; Figure S1: ROC curves for time to ROSC, age, and lactate on admission for unfavorable outcome in IHCA population; Table S3: Univariate correlation analysis for quantitative variables in out-of-hospital cardiac arrest population; Table S4: Univariate correlation analysis for categorial variables in OHCA population; Figure S2: ROC curves for time to ROSC, age, and lactate on admission for unfavorable outcome in OHCA population; Table S5: Univariate correlation analysis for quantitative variables in overall cohort; Table S6: Univariate correlation analysis for categorial variables in overall cohort; Table S7: Multivariate logistic regression for overall cohort; Figure S3: ROC curve for generalized logistic model for overall population.
