# Supplementary material for: A Comparison of Prognostic Factors in a Large Cohort of In-Hospital and Out-of-Hospital Cardiac Arrest Patients
Source: Life (Basel). 2024 Mar 18;14(3):403. doi: 10.3390/life14030403 (PMC10971552; doi:10.3390/life14030403)
Supplement: Supplementary file 1 [file life-14-00403-s001.zip › life-2887016-supplementary.pdf]

Table S1. Univariate correlation analysis for quantitative variables in in-of-hospital cardiac arrest population.

Variable	Outcome at 3 months		P-value
	Good	Bad	
**n**	168	372	
**Age (years)**	61.7 (16.2)	63.7 (15.3)	0.003
**Epinephrine (mg)**	2 [1-3]	3 [1 – 5]	<0.0001
**Lactate on admission (mmol/L)**	4.6 [3.2 - 6.9]	6.4 [3.3 - 10.0]	<0.0001
**Time to ROSC (min)**	8 [4 – 15]	15 [9 – 24]	<0.0001
**Weight (Kg)**	80.5 (18.9)	78.2 (18.8)	0.29

One way ANOVA was used for normally distributed variables (Age and Weight): mean (s.d.) was reported. The non-parametric Mann Whitney U test was used for non-normally distributed variables: median [IQR] was reported.

Table S2. Univariate correlation analysis for categorial variables in IHCA population.

Units of measure	Factor	Group	Outcome at 3 months		X^2	df	P-value
			Good	Bad			
	n		168	372			
n (%)	Witnessed Arrest	0	17 (10.1)	66 (17.7)	4.6	1	0.032
		1	151 (90.0)	306 (82.3)			
n (%)	AKI	0	86 (51.2)	134 (36)	0.03	1	0.001
		1	82 (48.8)	238(64)			
n (%)	Bystander CPR	0	16 (9.5)	77 (20.7)	9.4	1	0.002
		1	152 (90.5)	295 (79.3)			
n (%)	Chronic Heart Failure	0	119 (70.8)	246 (66.1)	0.96	1	0.32
		1	49 (29.2)	126 (33.9)			
n (%)	Chronic Kidney Disease	0	130 (77.4)	267 (71.8)	1.6	1	0.21
		1	38 (22.6)	105 (28.2)			
n (%)	COPD	0	135 (80.4)	299 (80.4)	0	1	1
		1	33 (19.6)	73 (19.6)			
n (%)	Coronary artery disease	0	102 (60.7)	240 (64.5)	0.57	1	0.45
		1	66 (39.3)	132 (35.5)			

n (%)	Steroids	0	130 (77.4)	265 (71.2)	1.9	1	0.17
		1	38 (22.6)	107 (28.8)			
n (%)	CRRT	0	137 (81.5)	283 (76.1)	1.7	1	0.19
		1	31 (18.5)	89 (23.9)			
n (%)	Diabetes	0	119 (70.8)	260 (69.9)	0.014	1	0.90
		1	49 (29.2)	112 (30.1)			
n (%)	ECMO	0	156 (92.9)	334 (89.8)	0.96	1	0.33
		1	12 (7.1)	38 (10.2)			
n (%)	ECPR	0	153 (91.1)	341 (91.7)	3.7	1	0.15
		1	12 (8.9)	30 (8.3)			
n (%)	Haemorragic Events	0	159 (94.6)	328 (88.2)	4.8	1	0.029
		1	9 (5.4)	44 (11.8)			
n (%)	HIV	0	166 (98.8)	370 (99.5)	0.077	1	0.78
		1	2 (1.2)	2 (0.5)			
n (%)	Hypertension	0	83 (49.4)	203 (54.6)	1	1	0.31
		1	85 (50.6)	169(45.4)			
n (%)	Arterial Hypothermia	0	96 (57.1)	184 (49.5)	2.4	1	0.12
		1	72 (42.9)	188 (50.5)			
n (%)	IABP	0	157 (93.5)	355 (95.4)	0.56	1	0.45
		1	11 (6.5)	17 (4.6)			
n (%)	Dobutamine	0	105 (62.5)	201 (54)	3	1	0.08
		1	63 (37.5)	171 (46)			
n (%)	Vasopressors	0	51 (30.4)	68 (18.3)	9.1	1	0.002
		1	117 (69.6)	304 (81.7)			
n (%)	Liver Cirrhosis	0	164(97.6)	340 (91.4)	6.2	1	0.012
		1	4 (2.4)	32 (8.6)			
n (%)	Male Sex	0	63 (37.5)	139 (37.4)	0	1	1
		1	105 (62.5)	233 (62.6)			
n (%)	Previous Neurological Disease	0	148 (88.1)	299 (80.4)	4.3	1	0.038
		1	20 (11.9)	73 (19.6)			
n (%)	Cause of cardiac arrest	Cardiac	96 (57.1)	160 (43)	8.7	1	0.003
		Non-cardiac	72 (42.9)	212 (57)			
n (%)	Percutaneous Coronary Intervention	0	145 (86.3)	358 (96.2)	16	1	0.0001
		1	23 (13.7)	14 (3.8)			
n (%)	Presentation Rhythm	Non-shockable	72 (42.9)	260 (69.9)	38	2	<0.0001 #
		Shockable	88 (52.4)	95 (25.5)			
		Unkown	8 (4.7)	17 (4.6)			
n (%)	Shock	0	96 (57.1)	146 (39.2)	14	1	<0.001
		1	72 (42.9)	226 (60.8)			

n (%)	Presentation Rhythm	Asystole	35 (20.8)	171 (46)	44	3	<0.0001#
		Shockable-Rhythm	88 (52.4)	95 (25.5)			
		PEA	37 (22)	89 (23.9)			
		Unkown	8 (4.8)	17 (4.6)			

Pearson X^2 test was used for comparison between two groups; # Overall p-value (adjustment method: Bonferroni). HIV: human immunodeficiency virus; CRP: cardiopulmonary resuscitation; PEA: pulseless electrical activity; ROSC: return to spontaneous circulation; CRRT: Continuous Renal Replacement Therapy; ECMO: extracorporeal membrane oxygenation; ECPR: extracorporeal cardiopulmonary resuscitation; IABP: Intra-Aortic Balloon Pump; ICU: intensive Care Unit; AKI: acute kidney insufficiency; COPD: chronic obstructive pulmonary disease.

Figure S1. ROC curves for time to ROSC, age and lactate on admission for Unfavourable Outcome in IHCA population

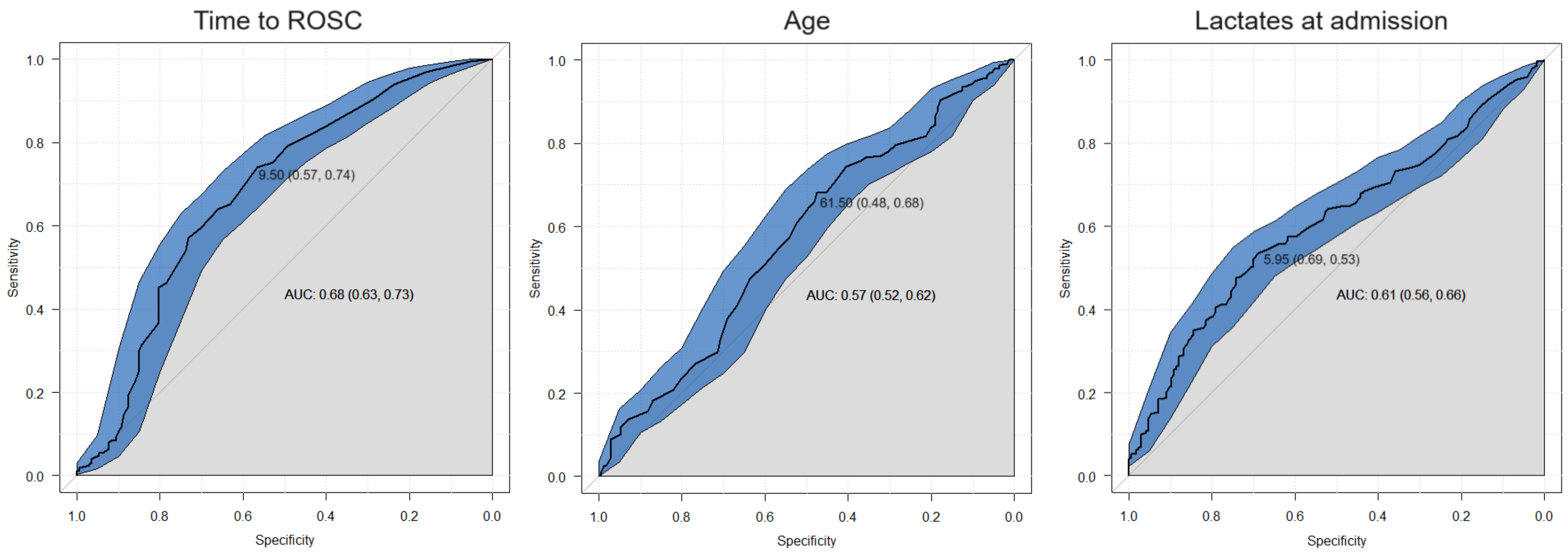

The optimal cut-off in IHCA population for time to ROSC, age and lactate on admission to predict UO were 9.5 min (Se 74%; Sp 57%; AUC 0.68 [95%CI 0.63-0.73]), 61.5 years (Se 68%, Sp 48%; AUC 0.57 [95%CI 0.52-0.63]) and 5.95 mmol/L (Se 53%, Sp 69%; AUC 0.61 [95%CI 0.56-0.66]), respectively.

Table S3. Univariate correlation analysis for quantitative variables in out-of-hospital cardiac arrest population.

Factor	Outcome at 3 months		P-value
	**Good**	**Bad**	
**n**	160	407	
**Age (years)**	57.66 (12.9)	63.70 (15.3)	<0.0001
**Epinephrine (mg)**	3 [1-5]	4 [2-7]	<0.0001
**Lactate on admission (mmol/L)**	4.5 [3.6 – 7.9]	7.1 [3.9 – 10.9]	<0.0001
**Time to ROSC (min)**	15 [10 – 25]	22 [15 – 30]	<0.0001
**Weight (kg)**	76.5 [70 – 83.3]	77 [65.3 – 89.0]	0.28

One way ANOVA was used for normally distributed variables (Age): mean (s.d.) was reported. The non-parametric Mann Whitney U test was used for non-normally distributed variables: median [IQR] was reported.

Table S4. Univariate correlation analysis for categorial variables in OHCA population.

Units of measure	Factor	Group	Outcome at 3 months		X^2	df	p-value
			**Good**	**Bad**			
	n		160	407			
n (%)	Witnessed Arrest	0	32 (20)	146 (35.9)	13	1	<0.001
		1	128 (80)	261 (64.1)			
n (%)	AKI	0	91 (56.9)	203 (49.9)	2	1	0.16
		1	69 (43.1)	204 (50.1)			
n (%)	Bystander CPR	0	68 (42.5)	224 (55)	6.7	1	0.009
		1	92 (57.5)	183 (45)			
n (%)	Chronic Heart Failure	0	133 (83.1)	338 (83)	0	1	1
		1	27 (16.9)	69 (17)			
n (%)	Chronic Kidney Disease	0	151 (94.4)	374 (91.9)	0.7	1	0.40
		1	9 (5.6)	33 (8.1)			
n (%)	COPD	0	139 (86.9)	330 (81.1)	2.3	1	0.10
		1	21 (13.1)	77 (18.9)			
n (%)	Coronary Artery Disease	0	99 (61.9)	262 (64.4)	0.21	1	0.65
		1	61 (38.1)	145 (35.6)			

n (%)	Steroids	0	140 (87.5)	326 (80.1)	3.8	1	0.051
		1	20 (12.5)	81 (19.9)			
n (%)	CRRT	0	147 (91.9)	367 (90.2)	0.23	1	0.63
		1	13 (8.1)	40 (9.8)			
n (%)	Diabetes	0	136 (85)	326 (80.1)	1.5	1	0.22
		1	24 (15)	81 (19.9)			
n (%)	ECMO	0	145 (90.6)	356 (87.5)	0.83	1	0.36
		1	15 (9.4)	51 (12.5)			
n (%)	ECPR	0	150 (93.7)	371 (91.2)	0.61	1	0.44
		1	10 (6.3)	36 (8.8)			
n (%)	Hemorragic Events	0	151 (94.4)	382 (93,9)	0.0014	1	0.97
		1	9 (5.6)	25 (6.1)			
n (%)	HIV	0	159 (99.4)	406 (99.7)	0.23	1	0.63
		1	1 (0.6)	1 (0.3)			
n (%)	Arterial Hypertension	0	100 (62.5)	250 (61.4)	0.02	1	0.89
		1	60 (37.5)	157 (38.6)			
n (%)							
	Hypothermia	0	35 (21.9)	112 (27.5)	1.6	1	0.20
		1	125 (78.1)	295 (72.5)			
n (%)	IABP	0	153 (95.6)	392 (96.3)	0.02	1	0.89
		1	7 (4.4)	15 (3.7)			
n (%)	Dobutamine	0	101 (63.1)	234 (57.5)	1.3	1	0.26
		1	59 (36.9)	173 (42.5)			
n (%)	Vasopressors	0	66 (41.2)	86 (21.1)	23	1	<0.0001
		1	94 (58.8)	321 (78.9)			
n (%)	Liver Cirrhosis	0	159 (99.4)	391 (96.1)	3.3	1	0.07
		1	1 (0.6)	16 (3.9)			
n (%)	Male Sex	0	40 (25)	137 (33.7)	3.6	1	0.057
		1	120 (75)	270 (66.3)			
n (%)	Previous Neurological Disease	0	146 (91.2)	339 (83.3)	5.3	1	0.022
		1	14 (8.8)	68 (16.7)			
n (%)	Cause of cardiac arrest	Cardiac	125 (78.1)	231 (56.8)	22	1	0.07
		Non-cardiac	35 (21.9)	176 (43.2)			
n (%)	Percutaneous Coronary Intervention	0	103 (64.4)	347 (85.3)	29	1	<0.0001
		1	57 (35.6)	60 (14.7)			
n (%)	Presentation Rhythm	Non-shockable	36 (22.6)	267 (65.6)	17	1	<0.0001
		Shockable	123 (76.8)	132 (32.4)			
		Unknown	1 (0.6)	8 (2)			
n (%)	Shock	0	114 (71.2)	211 (51.8)	17	1	<0.0001

		1	46 (28.8)	196 (48.2)			
n (%)	Presentation Rhythm	Shockable Rhythm	123 (76.8)	132 (32.4)	95	3	<0.0001#
		Asystole	26 (16.3)	209 (51.3)			
		PEA	10 (6.3)	58 (14.3)			
		Unkown	1 (0.6)	8 (2)			

Pearson X^2 test was used for comparison between two groups; # Overall p-value (adjustment method: Bonferroni). HIV: human immunodeficiency virus; CRP: cardiopulmonary resuscitation; PEA: pulseless electrical activity; ROSC: return to spontaneous circulation; CRRT: Continuous Renal Replacement Therapy; ECMO: extracorporeal membrane oxygenation; ECPR: extracorporeal cardiopulmonary resuscitation; IABP: Intra-Aortic Balloon Pump; ICU: intensive Care Unit; AKI: acute kidney insufficiency; COPD: chronic obstructive pulmonary disease.

Figure S2. ROC curves for time to ROSC, age and lactate on admission for Unfavourable Outcome in OHCA population

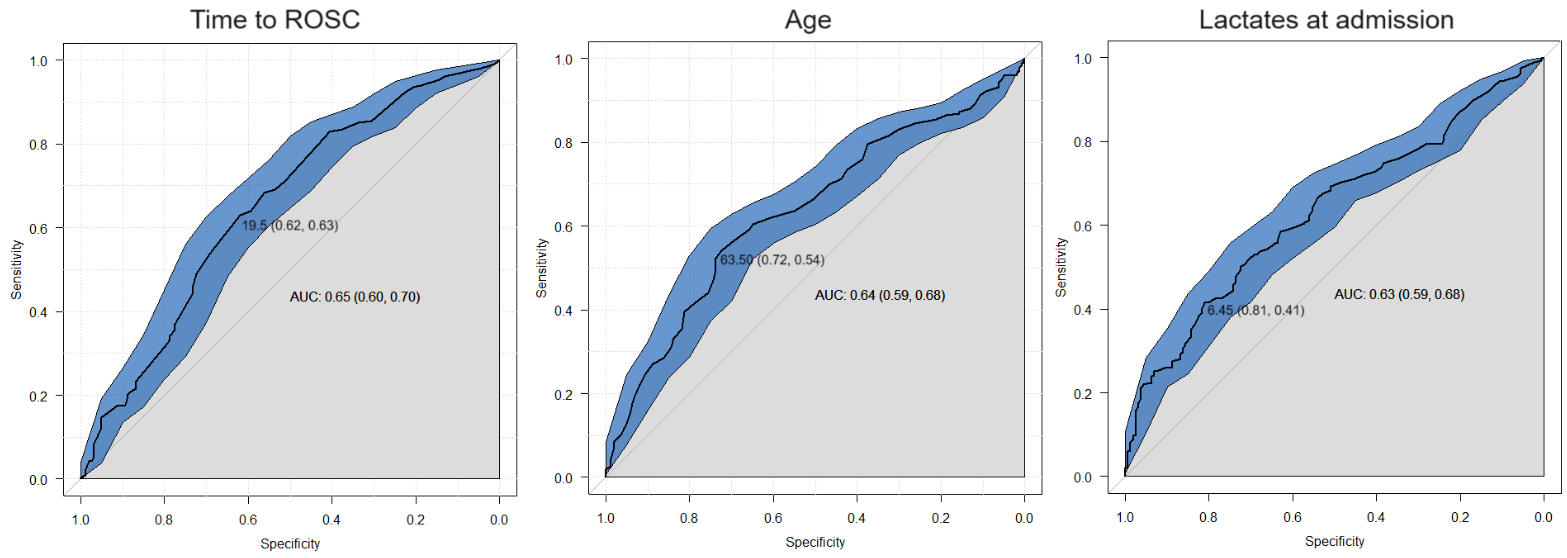

The optimal cut-off for OHCA population for time to ROSC, age, and lactate on admission to predict UO were 19.5 min (Se 63%; Sp62%; AUC 0.65 [95%CI 0.60-0.70]), 63.5 years (Se 54%, Sp 72%; AUC 0.64 [95%CI 0.59-0.68]) and 6.45 mmol/L (Se 41%, Sp 81%; AUC 0.63 [95%CI 0.58-0.68]), respectively.

Table S5. Univariate correlation analysis for quantitative variables in overall cohort.

Factor	Outcome at 3 months		P-value
	Good	Bad	
n	328	779	
**Age**	59.71 (14.76)	64.70 (15.01)	<0.0001
**Epinephrine (mg)**	2.00 [1.00-4.00]	3.00 [2.00, 6.00]	<0.0001
**Lactate on admission (mmol/L)**	4.55 [3.30, 7.27]	6.80 [3.70, 10.30]	<0.0001
**Time to ROSC (min)**	11.00 [5.00, 20.00]	20.00 [11.50, 28.00]	<0.0001
**Weight (kg)**	77.00 [68.00, 88.00]	77.00 [65.00, 89.00]	0.83

One way ANOVA was used for normally distributed variables (Age): mean (s.d.) was reported. The non-parametric Mann Whitney U test was used for non-normally distributed variables: median [IQR] was reported.

Table S6. Univariate correlation analysis for categorial variables in overall cohort

Units of measure	Factor	Group	Outcome at 3 months		X^2	df	P-value
			**Good**	**Bad**			
	n		328	779			
n (%)	Witnessed CA	0	49 (14.9)	212 (27.2)	19	1	<0.0001
		1	279 (85.1)	567 (72.8)			
n (%)	AKI	0	177 (54)	337 (43.3)	10	1	0.001
		1	151 (46)	442 (56.7)			
n (%)	Bystander CPR	0	84 (25.6)	301 (38.6)	17	1	<0.0001
		1	244 (74.4)	478 (61.4)			
n (%)	Chronic Heart Failure	0	252 (76.8)	584 (75)	0.34	1	0.56
		1	76 (23.2)	195 (25)			
n (%)	Chronic Kidney Disease	0	281 (85.7)	641 (82.3)	1.7	1	0.2
		1	47 (14.3)	138 (17.7)			
n (%)	COPD	0	274 (83.5)	629 (80.7)	1	1	0.31
		1	54 (16.5)	150 (19.3)			

n (%)	Coronary Artery Disease	0	201 (61.3)	502 (64.4)	0.86	1	0.35
		1	127 (38.7)	277 (35.6)			
n (%)	Steroids	0	270 (82.3)	591 (75.9)	5.2	1	0.02
		1	58 (17.7)	188 (24.1)			
n (%)	CRRT	0	284 (86.6)	649 (83.4)	1.5	1	0.22
		1	44 (13.4)	129 (16.6)			
n (%)	Diabetes	0	255 (77.7)	586 (75.2)	0.67	1	0.40
		1	73 (22.3)	193 (24.8)			
n (%)	ECMO	0	301 (91.8)	690 (88.6)	2.2	1	0.14
		1	27 (8.2)	89 (11.4)			
n (%)	ECPR	0	299 (93.1)	711 (91.5)	0.62	1	0.43
		1	22 (6.9)	66 (8.5)			
n (%)	Hemorragic Events	0	310 (94.5)	710 (91.1)	3.2	1	0.07
		1	18 (5.5)	69 (8.9)			
n (%)	HIV	0	325 (99.1)	776 (99.6)	0.42	1	0.52
		1	3 (0.9)	3 (0.4)			
n (%)	Arterial Hypertension	0	183 (55.8)	453 (58.2)	0.43	1	0.51
		1	145 (44.2)	326 (41.8)			
n (%)	Hypothermia	0	131 (39.9)	296 (38)	0.29	1	0.60
		1	197 (60.1)	483 (62.0)			
n (%)	IABP	0	310 (94.5)	747 (95.9)	0.72	1	0.39
		1	18 (5.5)	32 (4.1)			
n (%)	Dobutamine	0	206 (62.8)	435 (55.8)	4.3	1	0.038
		1	122 (37.2)	344 (44.2)			
n (%)	Vasopressors	0	117 (35.7)	154 (19.8)	31	1	<0.0001
		1	211 (64.3)	625 (80.2)			
n (%)	Liver Cirrhosis	0	323 (98.5)	731 (93.8)	9.9	1	0.002
		1	5 (1.5)	48 (6.2)			
n (%)	Male Sex	0	103 (31.4)	276 (35.4)	1.5	1	0.22
		1	225 (68.6)	503 (64.6)			
n (%)	Previous Neurological Disease	0	294 (89.6)	638 (81.9)	9.8	1	0.001
		1	34 (10.4)	141 (18.1)			
n (%)	Cause of cardiac arrest	Cardiac	221 (67.4)	391 (50.2)	27	1	<0.0001
		Non-cardiac	107 (32.6)	388 (49.8)			
n (%)	Site of Cardiac Arrest	IHCA	168 (51.2)	372 (47.8)	0.98	1	0.32
		OHCA	160 (48.8)	407 (52.2)			
n (%)	Percutaneous Coronary Intervention	0	248 (75.6)	705 (90.5)	42	1	<0.0001
		1	80 (24.4)	74 (9.5)			
n (%)	Presentation Rhythm	Non-shockable	108 (33)	527 (67.7)	120	1	<0.0001
		Shockable	211 (64.3)	227 (29.1)			

		Unknown	9 (2.7)	25 (3.2)			
n (%)	Shock	0	210 (64.0)	357 (45.8)	30	1	<0.0001
		1	118 (36.0)	422 (54.2)			
n (%)	Presentation Rhythm	Shockable	211 (64.3)	227 (29.1)	128	3	<0.0001#
		Asystole	61 (18.6)	380 (48.8)			
		PEA	47 (14.3)	147 (18.9)			
		Unkown	9 (2.7)	25 (3.2)			
n (%)	Age according to ROC threshold	<63.5	199 (60.7)	318 (40.8)	36	1	<0.0001
		≥ 63.5	129 (39.3)	461 (59.2)			
n (%)	Lactate on admission according to ROC threshold	<6.75	234 (71.8)	380 (49.6)	45	1	<0.0001
		≥ 6.75	92 (28.2)	386 (50.4)			
n (%)	Time to ROSC according to ROC threshold	< 13.5	188 (57.3)	230 (29.5)	75	1	<0.0001
		≥ 13.5	140 (42.7)	549 (70.5)			

Pearson X2 test was used for comparison between two groups; # Overall p-value (adjustment method: Bonferroni). HIV: human immunodeficiency virus; CRP: cardiopulmonary resuscitation; PEA: pulseless electrical activity; ROSC: return to spontaneous circulation; CRRT: Continuous Renal Replacement Therapy; ECMO: extracorporeal membrane oxygenation; ECPR: extracorporeal cardiopulmonary resuscitation; IABP: Intra-Aortic Balloon Pump; ICU: intensive Care Unit; AKI: acute kidney insufficiency; COPD: chronic obstructive pulmonary disease.

Table S7. Multivariate logistic regression for overall cohort.

	**Complete Logistic Multivariate Model**				**Final Model (Stepwise back-/forward based on AIC)**			
**VARIABLE**	**OR**	**IC95%**		**p-value**	**OR**	**IC95%**		**p-value**
**Witnessed CA [T.1]**	0.478	0.319	0.717	<0.001	0.491	0.330	0.731	<0.001
**Time to ROSC (min)**	1.030	1.020	1.050	<0.0001	1.030	1.020	1.050	<0.0001
**Site of Cardiac Arrest [OHCA]**	1.300	0.924	1.840	0.13	1.300	0.934	1.810	0.12
**Non-cardiac cause [T.1]**	1.790	1.280	2.510	<0.001	1.800	1.290	2.500	<0.001
**Previous Neurological disease [T.1]**	1.880	1.180	2.990	0.007	1.930	1.220	3.050	0.005
**Lactate on admission (mmol/L)**	1.090	1.040	1.140	<0.001	1.090	1.040	1.130	<0.001
**Liver Cirrhosis [T.1]**	3.000	1.100	8.210	0.032	3.020	1.110	8.160	0.03
**Presentation Rhythm [Shockable]**				#				#
**Presentation Rhythm [T.Asystole]**	4.920	3.410	7.100	<0.0001	4.910	3.410	7.060	<0.0001
**Presentation Rhythm [T.PEA]**	2.120	1.370	3.270	<0.001	2.140	1.390	3.290	<0.001
**Presentation Rhythm [T.Unknown]**	2.680	1.100	6.510	0.029	2.520	1.060	6.030	0.04
**Shock [T.1]**	1.770	1.260	2.490	0.001	1.800	1.310	2.470	<0.001
**Age (years)**	1.030	1.020	1.050	<0.0001	1.040	1.020	1.050	<0.0001

**Male Sex [T.1]**	0.899	0.647	1.250	0.52	-	-	-
**CRRT [T.1]**	0.895	0.551	1.460	0.655	-	-	-
**Chronic Kidney Disease [T.1]**	0.915	0.568	1.470	0.71	-	-	-
**HIV [T.1]**	0.409	0.068	2.470	0.33	-	-	-
**ECMO [T.1]**	0.932	0.519	1.680	0.81	-	-	-
**Steroids [T.1]**	1.060	0.714	1.580	0.77	-	-	-
**AKI [T.1]**	1.260	0.892	1.770	0.191	-	-	-
**Hemorragic Events[T.1]**	0.901	0.483	1.680	0.74	-	-	-
**Null deviance**				1330.7 on 1090 degrees of freedom			1331.4 on 1091 degrees of freedom
**Residual deviance**				1034.8 on 1069 degrees of freedom			1039.9 on 1079 degrees of freedom
**AIC**				1079			1066
**Hosmer and Lemeshow goodness of fit (GOF)**				χ^2 = 7.5, df = 8, p-value = 0.5			χ^2= 10, df = 8, p-value = 0.2
**AUC Model's ROC curve**				0.807 [95% CI 0.78 - 0.834]			0.805 [95% CI 0.777 - 0.832]

Complete regression analysis and final reduced model after stepwise back/forward selection based on Akaike Information Criterion (AIC). Overall p value for Presentation Rhythm: <0.0001. *L*onger time to ROSC, older age, higher lactate on admission, an unwitnessed CA, a *p*resentation non-shockable rhythm, a non-cardiac cause of CA, the occurrence of shock, the presence of previous neurological disease, the presence of liver cirrhosis were independent predictors of UO. HIV: human immunodeficiency virus; PEA: pulseless electrical activity; ROSC: return to spontaneous circulation; CRRT: Continuous Renal Replacement Therapy; ECMO: extracorporeal membrane oxygenation; AKI: acute kidney insufficiency.

Figure S3. ROC curve for Generalised Logistic Model for overall population

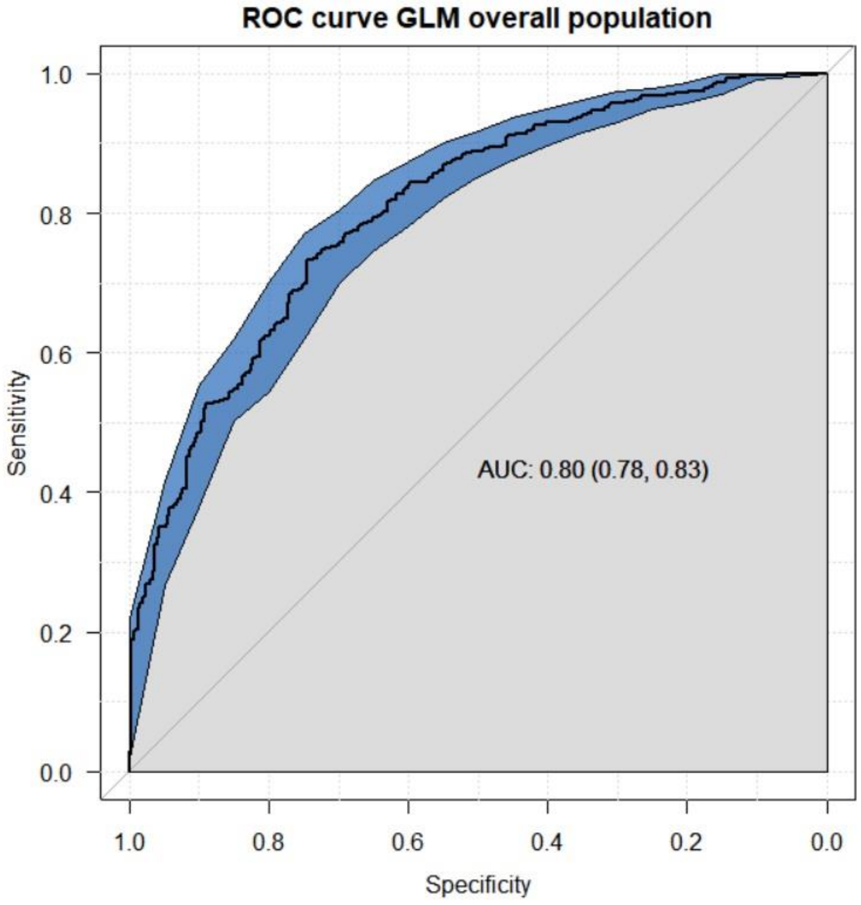

The ROC curve to assess the discriminatory ability of the model was 0.805 [95% CI 0.777 - 0.832].
